# Population genomics of the white-beaked dolphin (*Lagenorhynchus albirostris*): Implications for conservation amid climate-driven range shifts

**DOI:** 10.1038/s41437-024-00672-7

**Published:** 2024-02-01

**Authors:** Marc-Alexander Gose, Emily Humble, Andrew Brownlow, Dave Wall, Emer Rogan, Guðjón Már Sigurðsson, Jeremy J. Kiszka, Charlotte Bie Thøstesen, Lonneke L. IJsseldijk, Mariel ten Doeschate, Nicholas J. Davison, Nils Øien, Rob Deaville, Ursula Siebert, Rob Ogden

**Affiliations:** 1 Royal (Dick) School of Veterinary Studies and the Roslin Institute, University of Edinburgh, Edinburgh, UK; 2 Scottish Marine Animal Stranding Scheme, School of Biodiversity, One Health and Veterinary Medicine, College of Medical, Veterinary and Life Science, University of Glasgow, Glasgow, UK; 3 Irish Whale and Dolphin Group (IWDG), Kilrush, Ireland; 4 School of Biological, Earth & Environmental Sciences, University College Cork, Cork, Ireland; 5 Marine and Freshwater Research Institute, Hafnarfjörður, Iceland; 6 Institute of Environment, Department of Biological Sciences, Florida International University, North Miami, FL USA; 7 Fisheries and Maritime Museum, Esbjerg, Denmark; 8 Division of Pathology, Department of Biomolecular Health Sciences, Faculty of Veterinary Medicine, Utrecht University, Utrecht, the Netherlands; 9 Institute of Marine Research (IMR), Bergen, Norway; 10 Institute of Zoology, Zoological Society of London, London, UK; 11 Institute for Terrestrial and Aquatic Wildlife Research, University of Veterinary Medicine Hannover Foundation, Hannover, Germany

**Keywords:** Ecological genetics, Molecular ecology, Population genetics, Conservation biology

## Abstract

Climate change is rapidly affecting species distributions across the globe, particularly in the North Atlantic. For highly mobile and elusive cetaceans, the genetic data needed to understand population dynamics are often scarce. Cold-water obligate species such as the white-beaked dolphin (*Lagenorhynchus albirostris*) face pressures from habitat shifts due to rising sea surface temperatures in addition to other direct anthropogenic threats. Unravelling the genetic connectivity between white-beaked dolphins across their range is needed to understand the extent to which climate change and anthropogenic pressures may impact species-wide genetic diversity and identify ways to protect remaining habitat. We address this by performing a population genomic assessment of white-beaked dolphins using samples from much of their contemporary range. We show that the species displays significant population structure across the North Atlantic at multiple scales. Analysis of contemporary migration rates suggests a remarkably high connectivity between populations in the western North Atlantic, Iceland and the Barents Sea, while two regional populations in the North Sea and adjacent UK and Irish waters are highly differentiated from all other clades. Our results have important implications for the conservation of white-beaked dolphins by providing guidance for the delineation of more appropriate management units and highlighting the risk that local extirpation may have on species-wide genetic diversity. In a broader context, this study highlights the importance of understanding genetic structure of all species threatened with climate change-driven range shifts to assess the risk of loss of species-wide genetic diversity.

## Introduction

Understanding within-species connectivity and diversity is essential for informing conservation management and can help in assessing the impact of local extinctions for species-wide genetic variation (Palsbøll et al. 2007; Pavlova et al. 2017). In the marine environment, limitations to dispersal are more subtle than in terrestrial systems due to the scarcity of geophysical barriers. Nevertheless, marine species often display genetic structuring influenced by environmental conditions such as physiography, salinity, thermal niches, social structure, movement patterns, and behavioural specialisation (Craig and Herman 1997; Foote et al. 2011; Hoelzel 2009). Disentangling these patterns can be challenging, yet is critical for conservation management of species threatened with anthropogenic impacts and environmental shifts driven by global climate change (Palsbøll et al. 2007). The latter is particularly alarming for species inhabiting cold temperatures, as their available habitat is likely to shift under global warming (Louis et al. 2020; Pauls et al. 2013). This can have strong impacts on distribution, abundance, and species-wide genetic diversity. For example, when differentiated populations are present in areas subject to strong environmental change with limited availability to new suitable habitat, extirpation may result in the loss of a significant proportion of species-wide genetic diversity (Razgour et al. 2013). This, in turn, can negatively affect the ability of the species to adapt to future changes, as high genetic diversity is believed to be a major driver of positively selected mutations (Kardos et al. 2021). The risk of local extirpation, either driven by climate change or by direct anthropogenic impact, can only be accurately assessed when sufficient information on range-wide population structure of a species is available. As global recognition of the significance of genetic variation in biodiversity conservation grows and national and international bodies increasingly enforce commitments to protect ocean habitats (CBD 2022; DeWoody et al. 2021; United Nations 2015), there has never been a more urgent time to explore the connectivity of cold-water marine species to achieve appropriate conservation management strategies in response to challenges posed by global climate change.

**Fig. 1:**
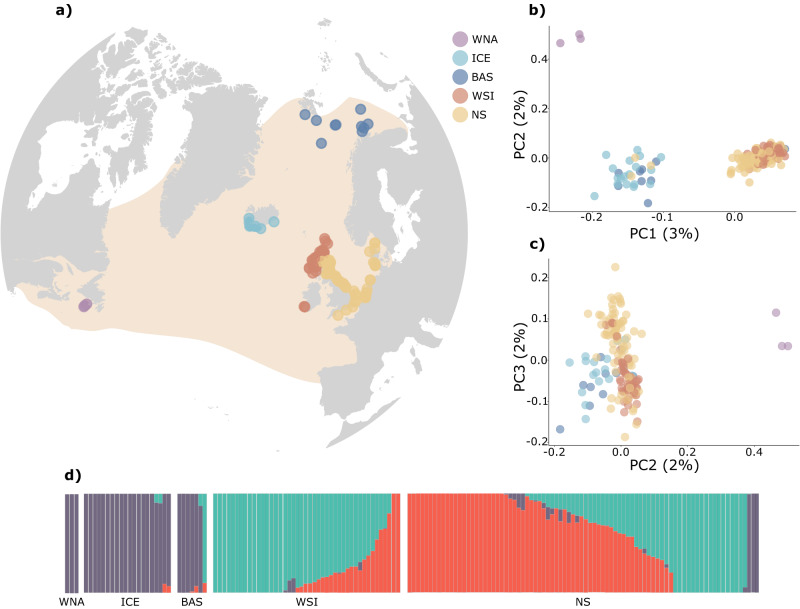
Genomic population structure of white-beaked dolphins across the North Atlantic Ocean and adjacent waters, based on 1092 SNPs. **a** Geographic locations of individual samples coloured by sampling location. The shaded area represents the distribution of the species across the North Atlantic. **b**, **c** Scatterplots displaying variation of the first two and the second and third principal components of a Principal Component Analysis (PCA). Percentage of variance for each axis shown in parentheses. **d** Admixture proportions of each individual for the most likely *K* (*K* = 3). ICE Iceland, BAS Barents Sea, NS North Sea, WSI western Scotland and Ireland, WNA western North Atlantic.

The white-beaked dolphin (*Lagenorhynchus albirostris*) is a cold-water obligate cetacean inhabiting continental shelf, shelf edge and continental slope waters of the temperate and sub-polar North Atlantic (Galatius and Kinze 2016). The species is common in the Canadian Atlantic, Greenland, Iceland, the Barents Sea, and parts of the North Sea and adjacent UK and Irish waters (Hammond et al. 2013; Hansen and Heide-Jørgensen 2013; Kinze et al. 2018; Lien et al. 2001; Øien 1996; Pike et al. 2019; Fig. 1a). In past decades a considerable northward shift in its southern distribution has been detected suggesting that white-beaked dolphins in the North Sea and adjacent UK waters avoid waters with higher sea surface temperatures (SSTs) (IJsseldijk et al. 2018; MacLeod et al. 2007; Waggitt et al. 2020). As SSTs in the North Sea are projected to further increase, more frequently exceeding the suitable threshold for white-beaked dolphins, the species risks facing a considerable northward-shift in this region (Dieterich et al. 2019; Evans and Waggitt 2020; Johns et al. 2003; Lambert et al. 2014). Additionally, the species faces numerous direct anthropogenic pressures, such as bycatch in commercial fisheries (Reeves et al. 2013), local unregulated harvesting (Takekawa 2000; Piniarneq 2021), prey depletion (Jackson et al. 2001), anthropogenic noise and chemical contaminants (Stone and Tasker 2006; Galatius, Bossi et al. 2013; Williams et al. 2023). In order to understand the consequences of predicted habitat shifts and other threats, it is necessary to investigate how white-beaked dolphins are connected across their range. As of now, morphometric studies have described differences in skull characteristics between the western North Atlantic and the North Sea indicative of separate populations (Mikkelsen and Lund 1994), suggesting some level of population structure. This was confirmed by genetic studies, supporting a distinction between northeast and northwest Atlantic populations, but also within the northeast Atlantic (Banguera-Hinestroza et al. 2010). Fernández et al. (2016) generated a panel of genome-wide SNPs, yet the study lacks an assessment of within-species genetic structure. For the conservation management of the white-beaked dolphin, a comprehensive assessment of population structure is needed to delineate more appropriate management units, as highlighted within the Agreement on the Conservation of Small Cetaceans of the Baltic, Northeast Atlantic, Irish and North Seas (ASCOBANS; ASCOBANS 2019). Here, we explore species-wide population structure, genetic diversity and contemporary geneflow of the white-beaked dolphin across the North Atlantic with the aim of providing guidance for improved conservation management of the species.

## Methods

### Sampling and DNA extraction

**Fig. 2:**
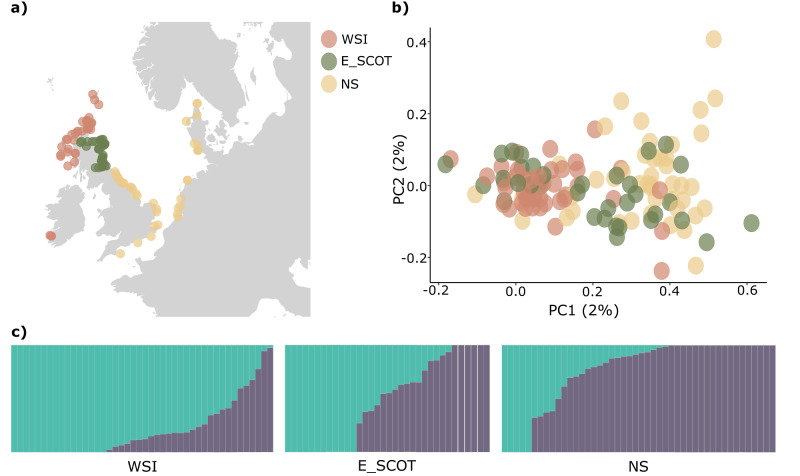
Investigation of a contact zone in the southern range of the white-beaked dolphin. **a** Geographic positions of the sampled individuals. The colours correspond to the two differentiated populations in the North Sea (NS) and western Scotland and Ireland (WSI), with the eastern Scottish (E_SCOT) individuals coloured separately. **b** Scatterplots of genetic structure from the first and the second axes of a Principal Component Analysis (PCA). Percentage of variance for each axis shown in parentheses. **c** Admixture proportions of the three assessed regions for *K* = 2.

A total of 169 tissue samples were obtained from preexisting archives and consisted of stranded (*n* = 133), by-caught (*n* = 23) or biopsied (*n* = 13) white-beaked dolphins sampled between 1992 and 2021. Prior to any further processing, the tissue samples were stored either dry frozen or in a ≥80% ethanol solution at −80 °C. The geographical distribution of the samples ranged from eastern Canada in the western North Atlantic (*n* = 3; biopsies), Iceland in the central North Atlantic (*n* = 23; bycatch) and the Barents Sea (*n* = 10; biopsies), Scotland (*n* = 81; strandings), England (*n* = 24; strandings), Ireland (*n* = 3; strandings), Denmark (*n* = 4; strandings), Germany (*n* = 12; strandings), The Netherlands (*n* = 8; strandings), and France (*n* = 1; stranding) in the eastern North Atlantic or adjacent waters (Figs. 1a and 2a). Genomic DNA was extracted using the Maxwell PureFood & GMO Authentication kit on a Maxwell RSC extraction robot. DNA was quantified using a Qubit fluorometer and tested for high molecular weight DNA content on a 1% agarose gel. DNA concentrations were standardised across samples and subsequently, 166 samples and 18 duplicate samples were submitted for DArTseq™ (Diversity Arrays Technology, Canberra, Australia). The DArTseq™ assay involves complexity reduction using a pair of restriction enzymes and amplification of the fragments via PCR. The resulting library is then shotgun-sequenced on an Illumina HiSeq 2500, producing single-end sequenced reads with a length of 130 bp. Due to later acquisition, the three samples from eastern Canada were submitted to Azenta Life Sciences (Chelmsford, Massachusetts, United States) for Short-Read Non-Human Whole Genome Sequencing (WGS) on an Illumina NovaSeq to ensure coverage of the same markers retained by the DArTseq™ approach. The raw data produced by Azenta was paired-end shotgun-sequenced with a fragment size of 150 bp.

### Read processing and mapping

Single-end DArTseq™ reads were quality-checked using FastQC and barcodes were trimmed using the *process_radtags* function within STACKS v2.5.4 (Andrews 2010; Catchen et al. 2013). Similarly, for the paired-end WGS reads FastQC was used for quality control and Trim Galore v0.6.6 was used to remove Illumina adapters. Both the DArTseq™ and WGS reads were mapped to the chromosomal-level genome assembly of *Lagenorhynchus albirostris* (Accession number: GCA_949774975.1) using the *bwa mem* function in BWA v0.7.17 (Li 2013). The output was assessed for mapping percentage and written to bam files using samtools v1.9 (Li et al. 2009).

Following this, the mapped files were sorted (*SortSam*) and all reads were assigned to a read group (*AddOrReplaceReadGroups*) using Picard Tools (Broad Institute 2019). Additionally, duplicates which can arise during library preparation were tagged and removed (*MarkDuplicates*) in the WGS reads. The output was indexed, and depth of coverage was calculated using samtools.

### Variant calling

Analysis of Next Generation Sequencing Data (ANGSD) software was used to detect variants and calculate genotype likelihoods across the 169 samples (Korneliussen et al. 2014).

An initial variant calling step was performed on all samples using base call and mapping quality filters (-*minMapQ* 30, -*minQ* 30, -*SNP_pval* 1e^−6^) and the output was written to PLINK format by specifying the *-doPlink* flag, which translates the variants to called genotypes. This initial dataset was inspected for levels of missing data and distribution of heterozygosity using the *--het* and --*missing* functions within PLINK v.1.09 (Purcell et al. 2007). Seven samples showed missing data ≥ 30% and three samples displayed above-average heterozygosity suggesting cross-contamination issues. These samples were removed from the workflow. Furthermore, pairwise relatedness between individuals was calculated by combining output from the PLINK *--genome* function and output from the programme NGSrelate (Korneliussen and Moltke 2015). Two pairs of samples showed a pairwise relatedness coefficient (*PI_HAT*) above 0.5 corresponding to first-degree relatedness (parent-offspring or full-siblings) and the sample with the lower genotyping rate of each pair was removed from subsequent analyses (Supplementary Fig. S1). The dataset for investigation of population structure thus comprised 157 individuals and the genotype likelihood calculation in ANGSD was repeated with additional filters on read depth (-*setMinDepth 785*, -*setMaxDepth 3140*) corresponding to a minimum depth of coverage of 5X and a maximum depth of coverage of 20X per locus per individual to avoid potential bases arising from sequencing errors following recommendations by O’Leary et al. (2018). Furthermore, we identified variants that were located in an interspersed repeat region using the programme RepeatMasker and excluded those from the variant calling by specifying the remaining sites using the *-sites* flag in ANGSD.

Further filtering of the multilocus genotypes was conducted in PLINK using a minor allele count of 2 to remove variants generated through uncertainties in base calling during sequencing. We examined the patterns of linkage disequilibrium decay in our data and observed a relatively steep decline in linkage disequilibrium in the initial portion of your linkage disequilibrium decay graph drawn by the programme ngsLD (Fox et al. 2019). This suggests stronger linkage patterns among nearby SNPs and therefore, we used the --*indep* function in PLINK to prune loci affected by linkage disequilibrium with a window size of 50 kb, a step size of 5 and a variant inflation factor of 2. The final dataset comprised 1092 Single Nucleotide Polymorphisms (SNPs) for all downstream population genetic analyses.

### Population structure

We investigated population structure using a number of different approaches. First, we performed a Principal Components Analysis (PCA) in PCAngsd (Meisner and Albrechtsen 2018). PCA is a dimensionality reduction approach, summarising genetic variation into Principal Components (PCs), which can be projected into axes to visualise genetic clustering. This approach is not influenced by geographic information. Eigenvalues of the first 20 PCs were inferred from the covariance matrix generated by PCAngsd. In R, population structure was visualised by plotting PCs one and two and PCs two and three (R Core Team 2022). To investigate patterns of fine-scale and sex-mediated population structure, the PCA was also performed with putative population assignments based on sampling site and for each sex separately, respectively. Second, *K*-means clustering was conducted to estimate the number of ancestral populations in the dataset using a maximum likelihood approach in NGSadmix (Skotte et al. 2013) and a Bayesian approach in Structure both with and without a-priori population assignments. A-priori assignments were informed by the clustering retained from the PCA. We investigated the most likely number of genetic clusters present in the dataset by calculating both DeltaK and Log Likelihood from the NGSadmix output and DeltaK using the Evanno method from the Structure output (Evanno et al. 2005; Supplementary Figs. S2 and S3). We examined the fit of the admixture proportions derived from the NGSadmix algorithm to its model assumptions by correlating the residual differences between called and predicted genotypes with the EvalAdmix software (Garcia-Erill and Albrechtsen 2020; Supplementary Fig. S4). The individual admixture proportions for each *K* and the correlation of residuals were plotted in R.

Finally, in order to investigate the significance of genetic population structure in the dataset, we grouped the samples into the populations informed by the approaches above and calculated the Weir and Cockerham pairwise fixation index (*F*_*ST*_) with 10,000 bootstraps using functions embedded in the *DartR* package (Weir and Cockerham 1984; Mijangos et al. 2022). Additionally, we grouped the samples by sampling sites (i.e., by country) and calculated pairwise *F*_*ST*_ to investigate patterns of fine-scale population structure.

### Contemporary gene flow

We estimated the proportion and direction of contemporary geneflow between genetic populations and between sampling sites using the BA3-SNPS extension of the software BayesAss, which enables computation of large genomic datasets (Mussmann et al. 2019; Wilson and Rannala 2003). In the first instance, initial runs were performed using the BA3-SNPs-autotune function to determine the optimal combination of the mixing parameters *deltaM* (mixing parameter for migration rates), *deltaA* (mixing parameter for allele frequencies) and *deltaF* (mixing parameter for inbreeding coefficients). These parameters were set to *deltaM* = 0.1563, *deltaA* = 0.3250 and *deltaF* = 0.0500. Five separate runs of BA3-SNPS were performed on different seeds with 10,000,000 MCMC iterations and 1,000,000 burn-ins on sampling intervals of 1000. Chain convergence of the runs was assessed in R and significance of the retained migration rates was assessed by a 95% confidence interval calculated as mean migration rate ±1.96 x mean standard deviation (Supplementary Fig. S5). The proportion and directionality of geneflow between populations was visualised in R.

### Isolation by distance

We tested the correlation of geographic and genetic distance by performing redundancy analysis and an ANOVA test on Euclidean distance matrices of geographic distances (*km*) and pairwise fixation indices (*F*_*ST*_) calculated between sampling sites using the *vegan* package in R. To achieve this, we calculated the minimum marine distance between sampling sites using a workflow described in Assis et al. (2013). Genetic distances were transformed to a continuous scale as ${GD}=\frac{{F}_{{ST}}}{(1-{F}_{{ST}})}$ to allow for correlation with geographic distances. The correlation of geographic distances and corresponding fixation indices was subsequently visualised in R.

### Multilocus heterozygosity

To investigate variation in genetic diversity across populations, we calculated the multilocus heterozygosity (*MLH*) across the 1092 SNPs as described in Stoffel et al. (2016) per population using the package *InbreedR*. Based on the detected population structure in the dataset, we grouped the individuals into their corresponding populations. Additionally, we visualised *MLH* distribution across the entire sample set.

### Investigation of a contact zone

Upon initial inspection of the observed structure, each analysis was repeated within a more localised approach in the North Sea and adjacent UK and Irish waters, to investigate finer-scale structure and the putative existence of a region of strong admixture in east Scotland in greater detail. For calculation of fixation indices, estimation of migration rates and heterozygosity, this was achieved by removing the admixed individuals from east Scotland to retain unbiased estimates.

## Results

### Data quality

The percentage of reads that mapped to the reference genome was 100% in almost all the samples. The mean coverage of all covered regions in the genome across all DArTseq^™^ samples was 12.75X and across the three WGS samples it was 8–10X across the entire genome. The initial number of variants detected in the unfiltered dataset was 542,232 SNPs across 169 individuals, which was reduced to 1092 highly informative SNPs across 157 individuals. Comparison of the 18 duplicate pairs ensured no genotyping errors were present our analyses.

### Population structure

We investigated the population structure present in the dataset using complexity reduction, maximum likelihood and Bayesian approaches combined with estimation of pairwise fixation indices and isolation-by-distance analysis. Combining results from all analyses, we observed both significant broad-scale and fine-scale population structure across the range of the white-beaked dolphin.

Mapping genetic origin against sampling location shows a clear differentiation of geographically isolated populations into four genetic clusters (Fig. 1b, c). Samples collected in both Iceland and the Barents Sea were assigned to the same genetic clade with a clear separation from the British Isles and the North Sea along the first PC axis. Similarly, the three individuals sampled in eastern Canada (WNA) were separated further along PC1, forming a separate cluster (Fig. 1b). Interestingly, NGSadmix and STRUCTURE analysis did not identify the WNA samples as a separate genetic cluster and grouped them together with the Icelandic and Barents Sea samples (Fig. 1d, Supplementary Figs. S6 and S7). Finer structure could be identified with separation of white-beaked dolphins sampled around west Scotland and Ireland and the coastlines of the North Sea along the third PC axis (Fig. 1c). This was further corroborated by NGSadmix and Structure analyses (Fig. 1d, Supplementary Figs. S6 and S7). A subsequent assessment of finer-scale and sex-mediated structure by a separate PCA confirms the overall structure detected by the previous approaches, demonstrating no clear difference in population structure between male and female white-beaked dolphins, implying the absence of sex-mediated dispersal (Supplementary Fig. S8).

Based on these results, naming conventions for the genetic clusters are introduced as the following regions: Western North Atlantic (WNA), Iceland and Barents Sea (ICE&BAS), west Scotland and Ireland (WSI) and North Sea (NS). Additionally, based on the pattern observed between NS and WSI, subsequent analyses were also performed with the eastern Scottish (E_SCOT) individuals as a separate group to investigate the influence a potential contact zone in this region may have on the estimation of population genetic parameters (Fig. 2a). We find that the majority of individuals previously grouped with NS but clustering with WSI were indeed from eastern Scotland (Fig. 2b). Admixture proportions for the three regions separately further visualise that eastern Scotland seems to be a contact zone between individuals of the southern and central North Sea and individuals sampled in Ireland and the west coast of Scotland (Fig. 2c).

**Fig. 3:**
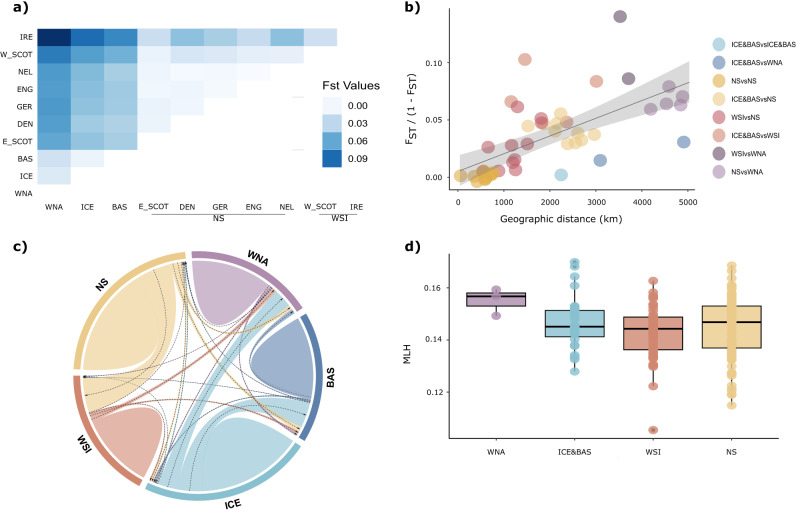
Fine-scale structure, isolation by distance, gene flow and genetic diversity in white-beaked dolphins. **a** Heatmap displaying pairwise Weir and Cockerham fixation indices (*F*_*ST*_) between ten sampled locations (France excluded due to small sample size). Darker shades of blue represent higher *F*_*ST*_ values and lighter shades represent lower *F*_*ST*_ values. **b** Relationship between geographic distance (km) and pairwise *F*_*ST*_ across all sampled regions (excluding France). The grey line represents the correlation and shaded area shows the 95% confidence interval. Points below line represent stronger connectivity and point above represent stronger differentiation than expected under a neutral IBD model. **c** Contemporary migration rates between five sampled regions as inferred by BayesAss analysis. Arrows are indicative of the direction of gene flow and width of bars represent the proportion of geneflow. **d** Multilocus heterozygosity (*MLH*) of each genetic population. Centre lines represent the median and the bound of the boxes extend from the first to the third quartile. The whiskers reflect the variability outside the interquartile range. Each point represents an individual sample. Region abbreviations for **a**: WNA Western North Atlantic, ICE Iceland, BAS Barents Sea, E_SCOT eastern Scotland, DEN Denmark, GER Germany, ENG England, NEL The Netherlands, W_SCOT western Scotland, IRE Ireland.

Pairwise fixation indices between the four populations retained by the PCA confirm that the samples from the western North Atlantic were significantly differentiated from the North Sea and western Scotland and Ireland samples (*F*_*ST_WNAvs.NS*_ = 0.05943055, *p* = 0.000; *F*_*ST_WNAvsWSI*_ = 0.078774106, *p* = 0.000), but displayed a lower, yet significant, differentiation to the Iceland and Barents Sea samples (*F*_*ST_WNAvs.ICE&BAS*_ = 0.01886975, *p* = 0.002). Similarly, the animals sampled in Iceland and the Barents Sea were significantly differentiated from the North Sea (*F*_*ST_ICE&BASvsNS*_ = 0.03946870, *p* = 0.000) and western Scotland and Ireland (*F*_*ST_ICE&BASvs.WSI*_ = 0.05496347, *p* = 0.000), while the latter two regions displayed a weak, but statistically significant differentiation (*F*_*ST_NSvs.WSI*_ = 0.006011101, *p* = 0.000). When excluding eastern Scottish samples, the fixation index between the North Sea and western Scotland and Ireland clades increases (*F*_*ST_NSvs.WSI*_ = 0.008685244, *p* = 0.000), indicative of a large proportion of admixed individuals from both populations in this region. The pairwise *F*_*ST*_ values calculated between sampling sites are visualised in Fig. 3a and exact values can be obtained from Supplementary Table S2.

### Contemporary gene flow

Estimation of the proportion and direction of geneflow between populations performed in BayesAss suggested little introgression from the western North Atlantic population into any of the other populations (Fig. 3c). Likewise, the Iceland and Barents Sea population and the two populations of the North Sea and western Scotland and Ireland showed very little evidence of gene flow in either direction. However, a higher proportion of gene flow was detected between Iceland and the Barents Sea and western North Atlantic populations in the direction of the western North Atlantic, specifically from Iceland to western North Atlantic with an estimated 0.166 migrants per generation (see Supplementary Table S1 for details). Similarly, there is a strong signal of unidirectional geneflow from Iceland into the Barents Sea (*m* = 0.222). Furthermore, a high level of introgression was detected between the two geographically neighbouring North Sea and western Scotland and Ireland populations with the majority of geneflow being facilitated by the North Sea population (*m* = 0.306). Interestingly, although the estimated migration rate remained similar when excluding eastern Scottish samples (*m* = 0.3265), the direction of geneflow reversed to a unidirectional influx from western Scotland and Ireland to the North Sea. All other migration rates did not exceed 0.06 migrants per generation and were therefore considered low.

### Isolation by distance

We investigated patterns of isolation by distance (IBD) by correlating geographic distance with pairwise fixation indices (genetic distance) between all sampling sites. The redundancy analysis (RDA) showed a moderate but statistically significant correlation between geographic and genetic distance (*r*^*2*^ = 0.3989931, *p* = 0.001). The IBD curve confirms that most datapoints fit within the confidence interval, but some points outside the general trend suggest deviations from IBD in both directions that is, stronger connectivity than expected under pure IBD (below curve) and stronger differentiation than expected under pure IBD (above curve; Fig. 3b).

### Genetic diversity

To assess genetic variation across the dataset, we calculated individual multilocus heterozygosity (*MLH*). We found *MLH* was normally distributed with a mean of 0.147 across all samples (min = 0.079, max = 0.173, Supplementary Fig. S9). When comparing *MLH* between genetic populations, we found little difference in heterozygosity between all sampled populations, but the western North Atlantic population displays slightly higher yet non-significant *MLH* compared to the other populations (Fig. 3d). The admixed eastern Scottish individuals did not inflate the heterozygosity estimates of the North Sea population as assessed by excluding these in a separate estimate (*MLH* = 0.1444 with E_SCOT vs. *MLH* = 0.1447 with E_SCOT removed).

## Discussion

Exploring the extent of genetic connectivity and differentiation across the range of the white-beaked dolphin is essential for conservation management, particularly given the numerous anthropogenic impacts on dolphin populations such as bycatch, accumulation of chemical contaminants and traditional hunts, as well as the putatively rapidly progressing effects of increasing SSTs threatening cold-water obligate species with habitat shifts. Using a combination of reduced representation and whole-genome sequencing, we investigated population structure, gene flow and genetic diversity in white-beaked dolphins from ten different sampling locations. We detect both broad-scale structure across the North Atlantic and fine-scale structure in the eastern North Atlantic and adjacent waters. The results of this study allow for a more informed delineation of management units for conservation and highlight the importance of population genomics in biodiversity conservation of species facing changes in their habitat amid global climate change.

Principal Components Analysis of 157 white-beaked dolphins detected a pattern of four differentiated clusters. Three of the four populations were also detected using *K*-means clustering approaches, but western North Atlantic samples were continuously grouped with Icelandic and Barents Sea individuals. This is likely due to limitations of the programmes Structure and NGSadmix to detect structure when gene flow is high and sample sizes are small (Waples and Gaggiotti 2006). The evaluation of the fit of our data to the admixture algorithm confirms that this method may not be able to disentangle the full ancestral history, likely due to gaps in sampling coverage across the species’ range and associated assumptions the algorithm makes based on the provided data set. Statistical evaluation of panmixia using Weir and Cockerham’s pairwise fixation indices reject the null hypothesis of continuous genetic connectivity between Iceland and Barents Sea and western North Atlantic, supporting the existence of four differentiated populations as shown in the PCA. We therefore conclude that *K* = 4 comprising of the western North Atlantic, Iceland and Barents Sea, North Sea, and west Scotland and Ireland, is the most likely number of populations in our dataset. Pairwise *F*_*ST*_ values were highest between western North Atlantic and west Scotland and Ireland and North Sea populations. Indeed, migration between these regions was very low in our data and a significant correlation of geographic and genetic distance suggests isolation by distance contributing largely to the observed differentiation. This is in agreement with previous genetic studies using mtDNA and microsatellite loci and a morphometric study describing distinct differences in skull characteristics between dolphins from the North Sea and the western North Atlantic (Banguera-Hinestroza et al. 2010; Mikkelsen and Lund 1994).

In direct contrast to the differentiation between the western North Atlantic and the North Sea, western Scotland and Ireland is the relatively strong connectivity between western North Atlantic and Iceland and Barents Sea populations, despite an apparent hiatus in distribution between the two latter areas (Pike et al. 2019). Remarkably, a complete homogeneity of genotypes from Iceland and the Barents Sea was found and is indicative of frequent (0.222 migrants per generation) long-distance individual migration events between the two regions as corroborated by our analysis on contemporary gene flow and in line with observed movement capabilities of the species (Rasmussen et al. 2013). Similarly, a relatively high migration rate (0.166 migrants per generation) was found between Iceland and the western North Atlantic, yet genetic distinction is persistent but weak. Abundance estimates from Iceland and the Barents Sea suggest large population sizes (Byrd et al. 2020; Øien 1996; Pike et al. 2019) which could be obscuring the presence of differentiation between two otherwise demographically separate clades (Waples 1998). Regular geneflow between populations and large population sizes could also contribute to the higher heterozygosity values in the western North Atlantic, though this could also be an artifact of different sequencing techniques in this population and the possibility of introducing batch effects in the estimates (Lou and Therkildsen 2022).

Within the eastern North Atlantic and adjacent waters, we detected a clear separation of Icelandic and Barents Sea white-beaked dolphins from individuals sampled around western Scotland and Ireland and the North Sea, as well as a regional separation of individuals sampled off the coast of the North Sea and those sampled off western Scotland and Ireland with a region of strong admixture in eastern Scotland. This result was in part anticipated and consistent with results from a previous study by Banguera-Hinestroza et al. (2010) who compared Barents Sea samples to the British Isles and North Sea. The introduction of samples from Iceland in our study gives a new dimension to the overall pattern of structure found in this species, as the minimal marine distance between sampling sites in Iceland and, for example, the Netherlands is comparable to the distance between Iceland and the Barents Sea (~2200 km) yet genetic distances and migration rates are in stark contrast. Hence, there is a strong implication that ecological factors could influence population structure in the eastern North Atlantic and adjacent waters. This is further supported by the consistent pattern of regional structure found between the North Sea and west Scotland and Ireland.

The region of strong admixture between white-beaked dolphins of the North Sea and of western Scotland and Ireland, located at the eastern Scottish coast, brings up interesting questions about the factors driving this pattern. Ecological differences may in part be responsible for the detected differentiation between the two neighbouring clades, and the occurrence of a contact zone could reflect a response to environmental change and resulting change in behaviour suggesting the contact zone is a recent phenomenon. An alternative explanation to the pattern could be a retrieval of a separate refugium population to the southern North Sea during the most recent LGM, which has been argued as a potential driver for regional structure in marine species of the North Atlantic (Hewitt 2000; Hoarau et al. 2007).

The limited understanding of white-beaked dolphin ecology, life history and habitat use hampers the interpretation of drivers of the observed population structure. Notably, seasonal migration from higher latitudes in the winter to lower latitudes in the summer have been observed in various regions (Canning et al. 2008; Fall and Skern-Mauritzen 2014; Pike et al. 2019). This could be influenced by numerous factors such as responses to migratory prey, site fidelity to certain areas during mating season, competition from other species or predator avoidance. Regarding diet, white-beaked dolphins have been reported to target higher level trophic gadoid fish with some regional variation across their range based on stomach content analyses (Dong et al. 1996; Jansen et al. 2010; Fall and Skern-Mauritzen 2014; Schick et al. 2020; Samarra et al. 2022). However, studies using stable isotopes show a clear preference for pelagic squids in the western North Atlantic versus a preference for higher trophic level fish in the eastern North Atlantic and Iceland (Samarra et al. 2022; Plint et al. 2023; Kiszka and Caputo, unpublished data). This difference may in part explain the elevated genetic distance that we found in our IBD analysis between the two sites of the North Atlantic. Within the eastern North Atlantic, seasonal occurrence during summer months has been argued to possibly result from site fidelity in both the North Sea and west Scotland and Ireland, potentially driving the fine-scale structure as observed in our study (Reeves et al. 1999; Canning et al. 2008; Brereton et al. 2013; Galatius, Jansen et al. 2013). Contrastingly, long-term photo ID monitoring of Icelandic dolphins suggest no strong signal of site fidelity (Bertulli et al. 2015). Altogether, further studies on white-beaked dolphin movement, diet, behaviour, and ecology of different populations are needed to explore potential drivers of the observed population structure and inform a more targeted management approach.

### Implications for conservation management

Our findings could have significant implications for conservation management at both regional and North Atlantic basin-wide scales by providing new evidence on fine-scale population structure of white-beaked dolphins. Currently, the species receives varying degrees of management; in the western North Atlantic, white-beaked dolphins are considered a single stock across their western range (Byrd et al. 2020). In Icelandic and Norwegian waters, the species receives no targeted management, while in its southern distribution it is managed as a single management unit (MU) comprising of the North Sea and the waters extending beyond the western coast of Ireland and the UK (IAMMWG 2015). Evans and Teilmann (2009) compiled all available information on the species and recommended four MUs comprising of the Labrador shelf, Icelandic waters, the Barents Sea and the North Sea and adjacent waters. Our results largely support this delineation, but based on our genetic analysis, our recommendations differ slightly.

The Labrador shelf population is only represented by three samples in our study from eastern Canada. These samples represent a differentiated clade in our analyses, generally supportive of the distinction of this region as a separate MU. However, a more in-depth assessment of structure is needed in this region, covering larger areas, and increasing sample size. An important aim for future studies will be the introduction of samples from Greenland, especially in the light of increasing rates of removals through traditional hunts and the uncertainty on the sustainability of these hunts (Piniarneq 2021).

Our findings also reveal strong connectivity between white-beaked dolphins sampled in Iceland and the Barents Sea, and strong differentiation between the Iceland and Barents Sea and the North Sea and west Scotland and Ireland. Populations in higher latitudes such as the Iceland and Barents Sea population are unlikely to experience habitat loss due to increasing SSTs and may in fact find more available habitat as sea ice retreats (Stafford et al. 2022). However, it may still be useful to assess white-beaked dolphins in these regions regarding their distribution, habitat use and behaviour as well as impact of anthropogenic activities to investigate their responses to potential environmental change and learn more about this populations’ ecology. It is recommended that the genetic connectivity between Iceland and the Barents Sea should be considered in future assessments of this population.

Most strikingly, our findings on fine-scale structure between the North Sea and western Scotland and Ireland warrant reconsideration of current local management (IAMMWG 2015). In this part of their range, white-beaked dolphins appear to strongly associate with SSTs below 12–13 °C (MacLeod et al. 2007) and therefore are likely to be especially vulnerable to increasing SSTs (Evans and Waggitt 2020). Furthermore, this region has been identified as a high- risk area for strong anthropogenic impact from climate change, pollution, and fishing (Davidson et al. 2012). Populations of white-beaked dolphins on the edge of their southern distribution are therefore likely to be impacted by climate-change associated habitat shifts (Lambert et al. 2014), in addition to numerous direct threats (Stone and Tasker 2006; Bearzi et al. 2006; Reeves et al. 2013; Galatius, Bossi et al. 2013; Williams et al. 2023). A recent northward-shift in their distribution based on strandings data (IJsseldijk et al. 2018; Williamson et al. 2021) and predictive habitat modelling (Lambert et al. 2014) is indicative of an ongoing contraction of suitable habitat around the British Isles and North Sea. The responses of the two local populations are difficult to predict. Possible scenarios range from a retreat to small pockets of suitable habitat, leading to small vulnerable populations, the total extirpation of the species in the area or a northward-shift and subsequently increased connectivity into waters currently occupied by dolphins of the genetically differentiated population around Iceland and the Barents Sea. Our assessment of local structure in this region indicates that eastern Scotland may currently be a contact zone for dolphins from the two southern clades, suggesting that further admixture could weaken the observed structure over time. Future genetic monitoring of these regions could help to predict how those populations may interact and what the genetic consequences could be. The potential risk of local extirpation of these two southern populations, and consequently the loss of a significant proportion of species-wide genetic diversity, should be emphasised in future management plans. Furthermore, formal assessment of the impact of factors that may cause additional mortality such as bycatch, pollution, and marine development should be a priority in future conservation efforts.

In a wider context, our study provides an example of the importance of assessing population genomics in marine species facing pressures from climate change and human impact. As the relevance of genetic diversity as a pillar of biodiversity conservation for long-term species survival gains acknowledgement from international and national policymakers (United Nations 2015; CBD 2022), detailed knowledge on the population structure and genetic variability is urgently needed. Using these data to understand the dynamics of these species can help in identifying vulnerable populations and assess the risk for the loss of species-wide genetic diversity by local depletion and continued human impact.

### Supplementary information

Supplementary Material

## Data Availability

Raw sequence reads are available at the European Nucleotide archive (ENA) under the accession number PRJEB71584.

## Code availability

All code for analyses and plotting can be accessed at https://github.com/MarcGose/WBD_PopGen.
