# Correction: Population genomics of the white-beaked dolphin (*Lagenorhynchus albirostris*): implications for conservation amid climate-driven range shifts

**DOI:** 10.1038/s41437-024-00699-w

**Published:** 2024-07-29

**Authors:** Marc-Alexander Gose, Emily Humble, Andrew Brownlow, Dave Wall, Emer Rogan, Guðjón Már Sigurðsson, Jeremy J. Kiszka, Charlotte Bie Thøstesen, Lonneke L. IJsseldijk, Mariel ten Doeschate, Nicholas J. Davison, Nils Øien, Rob Deaville, Ursula Siebert, Rob Ogden

**Affiliations:** 1 Royal (Dick) School of Veterinary Studies and the Roslin Institute, University of Edinburgh, Edinburgh, UK; 2 Scottish Marine Animal Stranding Scheme, School of Biodiversity, One Health and Veterinary Medicine, College of Medical, Veterinary and Life Science, University of Glasgow, Glasgow, UK; 3 Irish Whale and Dolphin Group (IWDG), Kilrush, Ireland; 4 School of Biological, Earth & Environmental Sciences, University College Cork, Cork, Ireland; 5 Marine and Freshwater Research Institute, Hafnarfjörður, Iceland; 6 Institute of Environment, Department of Biological Sciences, Florida International University, North Miami, FL USA; 7 Fisheries and Maritime Museum, Esbjerg, Denmark; 8 Division of Pathology, Department of Biomolecular Health Sciences, Faculty of Veterinary Medicine, Utrecht University, Utrecht, the Netherlands; 9 Institute of Marine Research (IMR), Bergen, Norway; 10 Institute of Zoology, Zoological Society of London, London, UK; 11 Institute for Terrestrial and Aquatic Wildlife Research, University of Veterinary Medicine Hannover Foundation, Hannover, Germany

**Keywords:** Ecological genetics, Molecular ecology, Population genetics, Conservation biology

Correction to: *Heredity* 10.1038/s41437-024-00672-7, published online 01 February 2024

In this article, the last two sentences in the Results section under "Contemporary geneflow" were incorrectly given as:

Interestingly, although the estimated migration rate remained similar when excluding eastern Scottish samples (*m* = 3265), the direction of geneflow reversed to a unidirectional influx from western Scotland and Ireland to the North Sea. All other migration rates did not exceed 0.06 migrants per generation and were therefore considered low.

But should have been:

Interestingly, although the estimated migration rate remained similar when excluding eastern Scottish samples (*m* = 0.3265), the direction of geneflow reversed to a unidirectional influx from western Scotland and Ireland to the North Sea. All other migration rates did not exceed 0.06 migrants per generation and were therefore considered low.

The given value for the estimated migration rate between the two mentioned groups was wrong. In the text it says *m* = 3265 when it should be *m* = 0.3265 instead.

The original article has been corrected.

