# Supplementary material for: Population genomics of the white-beaked dolphin (Lagenorhynchus albirostris): Implications for conservation amid climate-driven range shifts
Source: Heredity (Edinb). 2024 Feb 1;132(4):192–201. doi: 10.1038/s41437-024-00672-7 (PMC10997624; doi:10.1038/s41437-024-00672-7)
Supplement: Supplementary file 1 — Supplementary Material [file 41437_2024_672_MOESM1_ESM.pdf]

Supplementary Material

Population genomics of the white-beaked dolphin (*Lagenorhynchus albirostris*): Implications for conservation amid climate-driven range shifts

Marc-Alexander Gose, Emily Humble, Andrew Brownlow, Dave Wall, Emer Rogan, Guðjón Már Sigurðsson, Jeremy J. Kiszka, Charlotte Bie Thøstesen, Lonneke L. IJsseldijk, Mariel ten Doeschate, Nicholas J. Davison, Nils Øien, Rob Deaville, Ursula Siebert and Rob Ogden

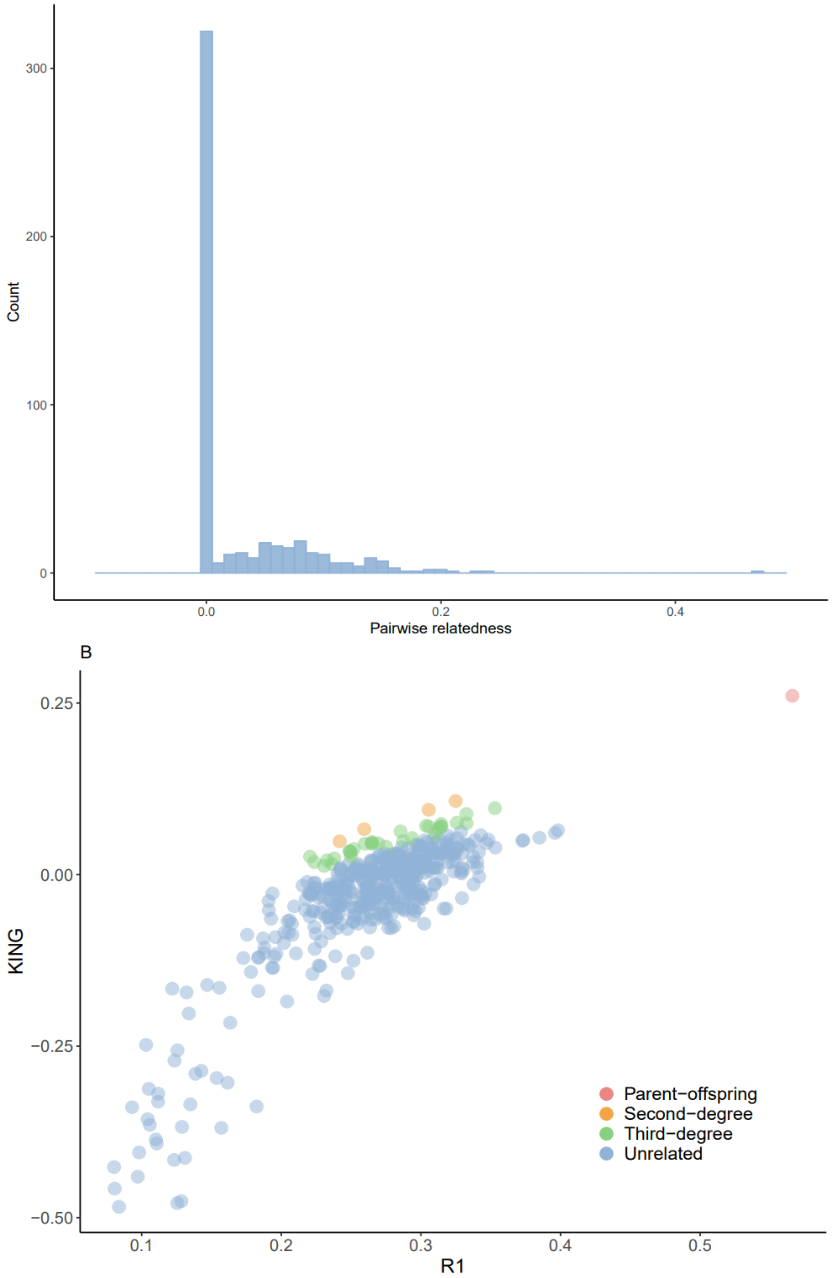

Figure S1: Genomic population-wide pairwise relatedness as exemplified by the ICE&BAS population. a) Distribution of the pairwise relatedness measure *PI_HAT* across a total of 496 comparisons. b) KING robust relatedness against R1 of pairwise comparisons between two individual samples. Classification of relatedness category based on pre-defined thresholds defined by Manichaikul et al. (2010).

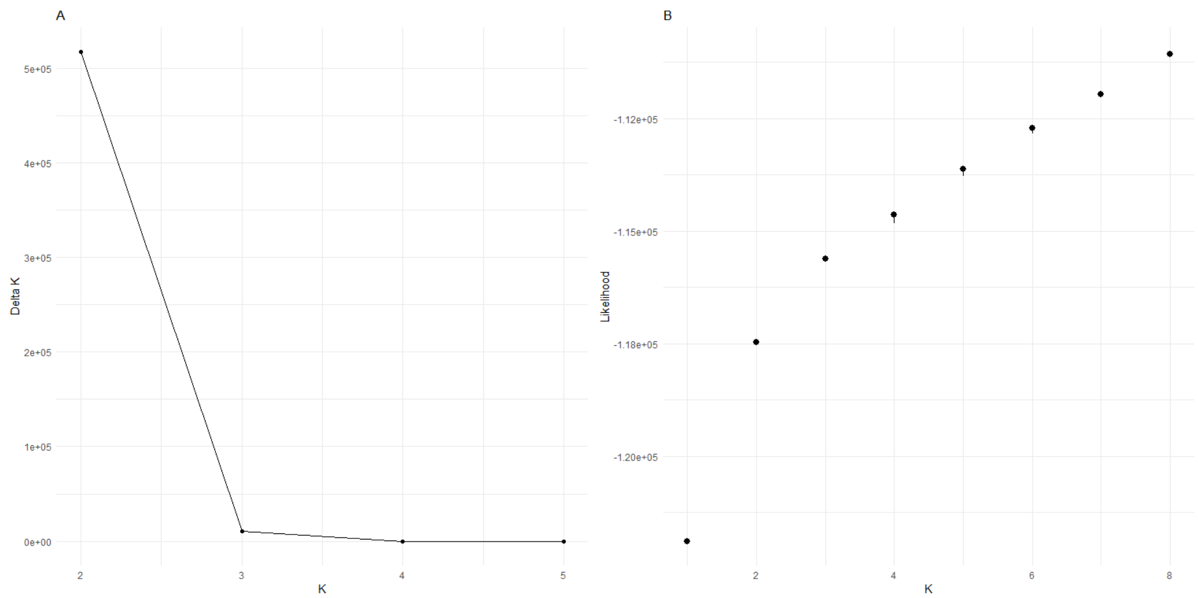

Figure S2: Delta K and Log Likelihood for NGSADMIX analysis.

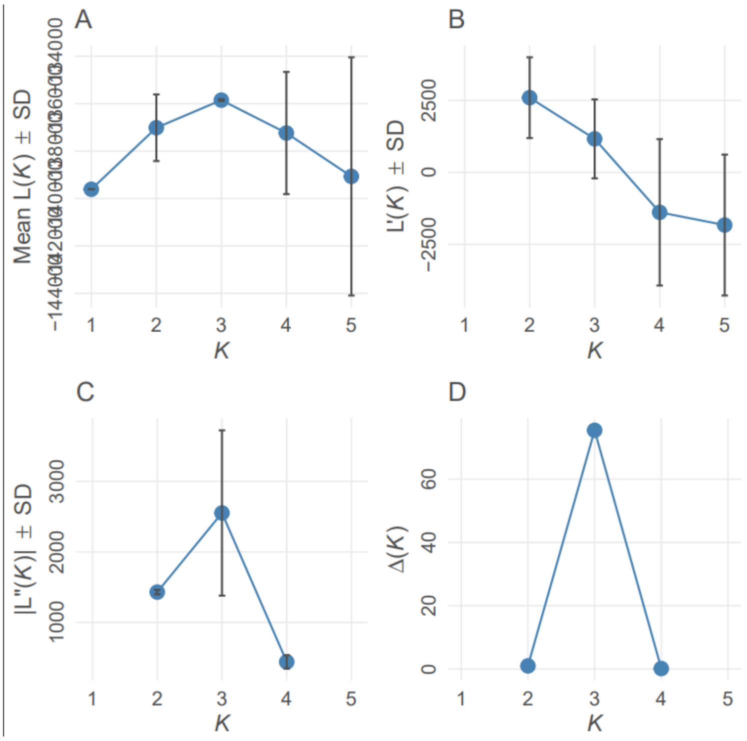

Figure S3: Delta K estimated by Evanno method from Structure output. A: The mean likelihood and variance per K. B: The mean rate of change of the likelihood distribution. C: The absolute of the second order rate of change of the likelihood per K value distribution. D: Delta K estimated as the mean of the absolute second order likelihood per K value divided by the standard deviation of the mean likelihood and variance of the K value.

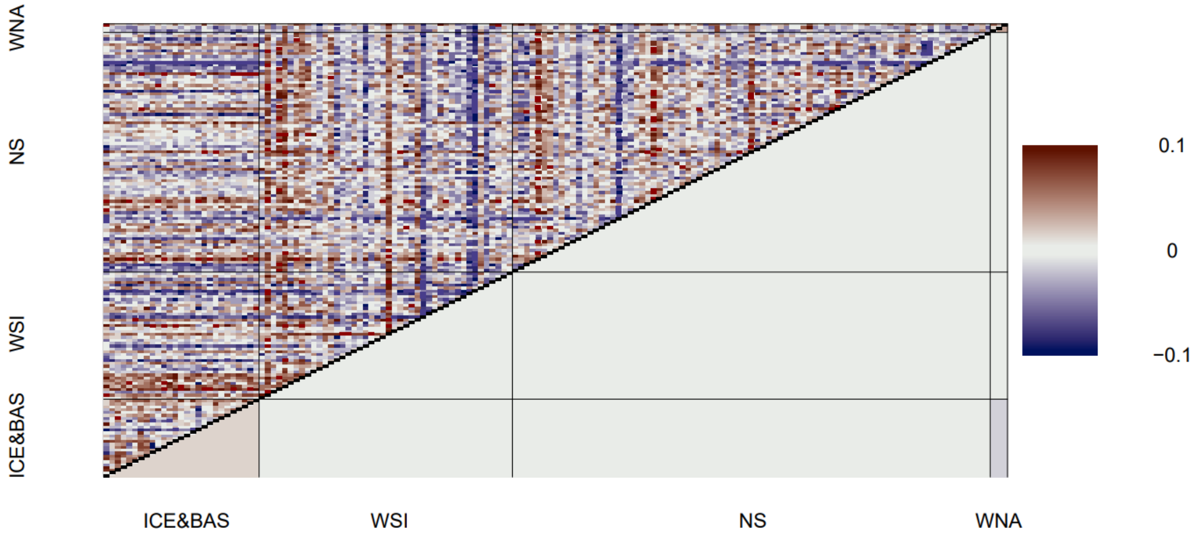

Figure S4: Evaluation of admixture proportions with *K*=3

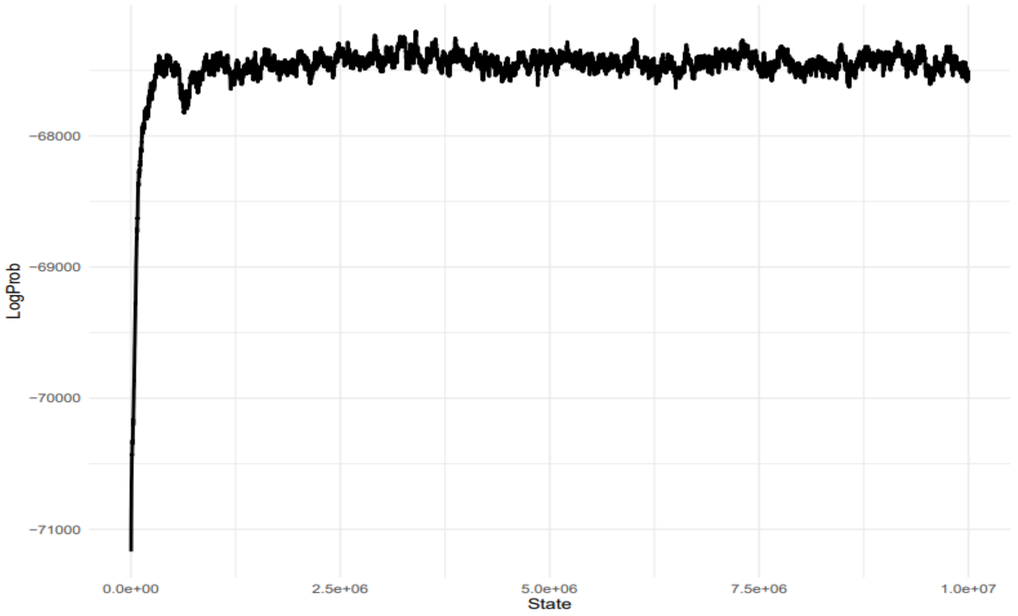

FigureS5: Profile of log probability traces for each iteration in the BayesAss MCMC analysis is depicted in BA3-SNPs. The BayesAss run comprised 10,000,000 iterations with a burn-in of 1,000,000 and a sampling interval of 1000. The log probability exhibits a sharp increase during the burn-in phase, followed by oscillations around a plateau, indicating convergence of the chain.

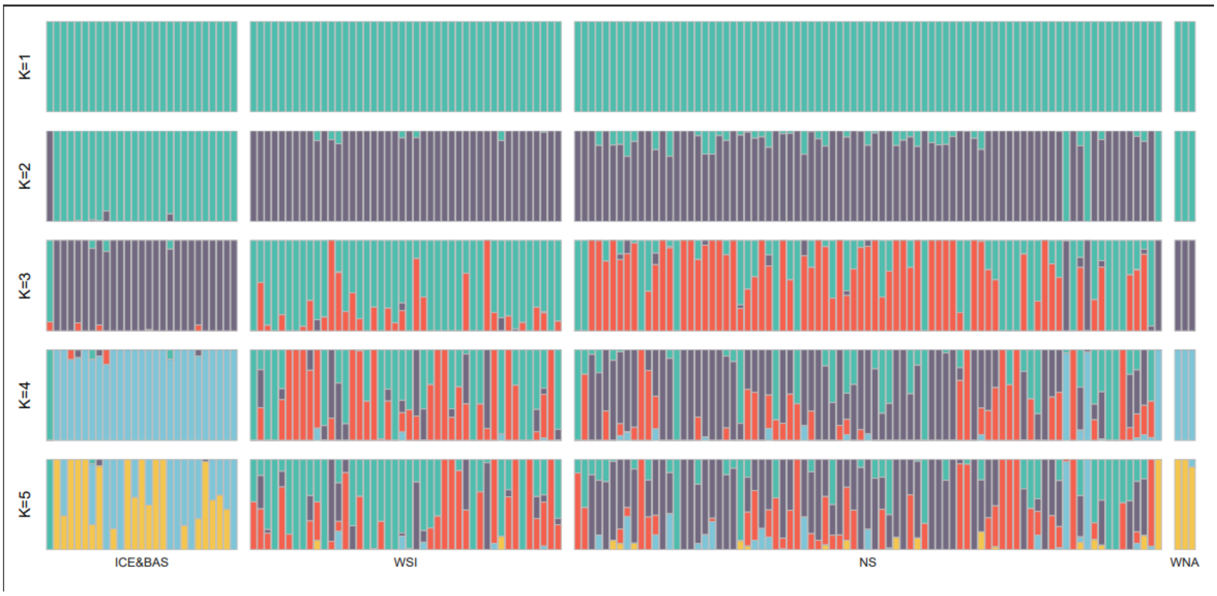

Figure S6: Admixture proportions across 157 individuals estimated by NGSADMIX for *K* = 1– 5.

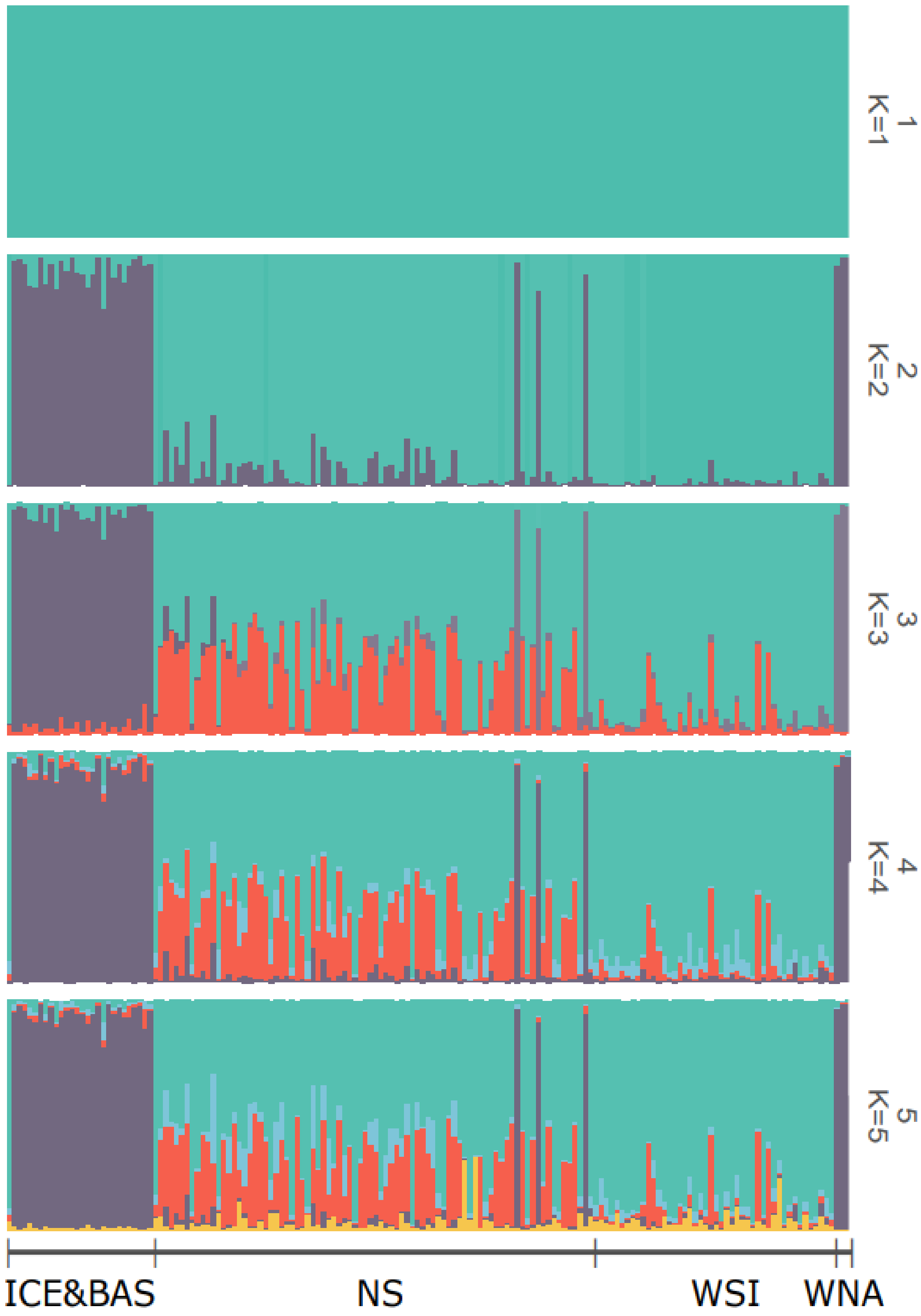

Figure S7: Admixture proportions across 157 individuals for K 1 – 5 estimated by STRUCTURE with information on population origin.

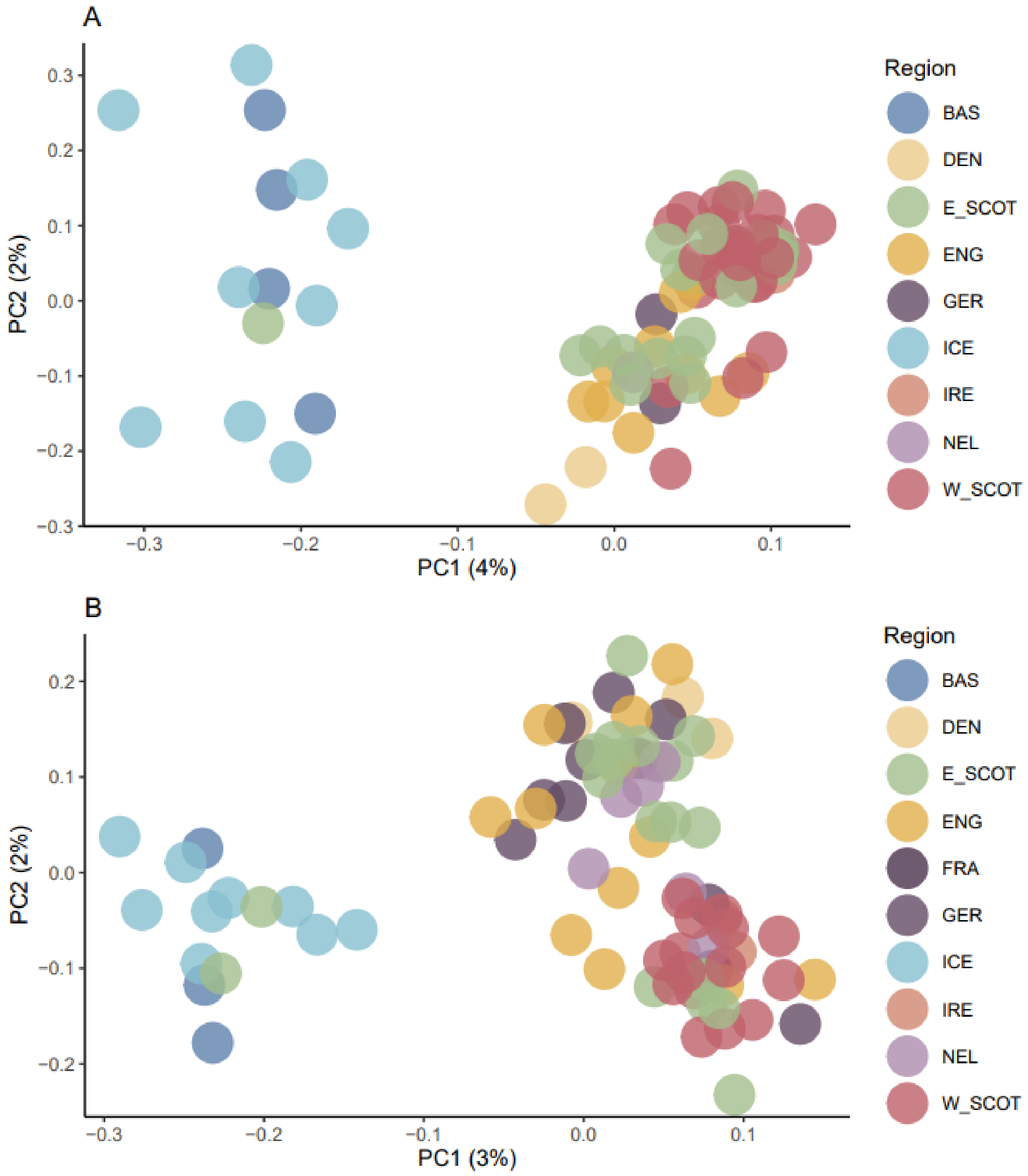

Figure S8: PC1&2 for all male samples (A) and all female samples (B) excluding WNA with a separation by sample site (Region). Percentage of variance of each axis are shown in parenthesis.

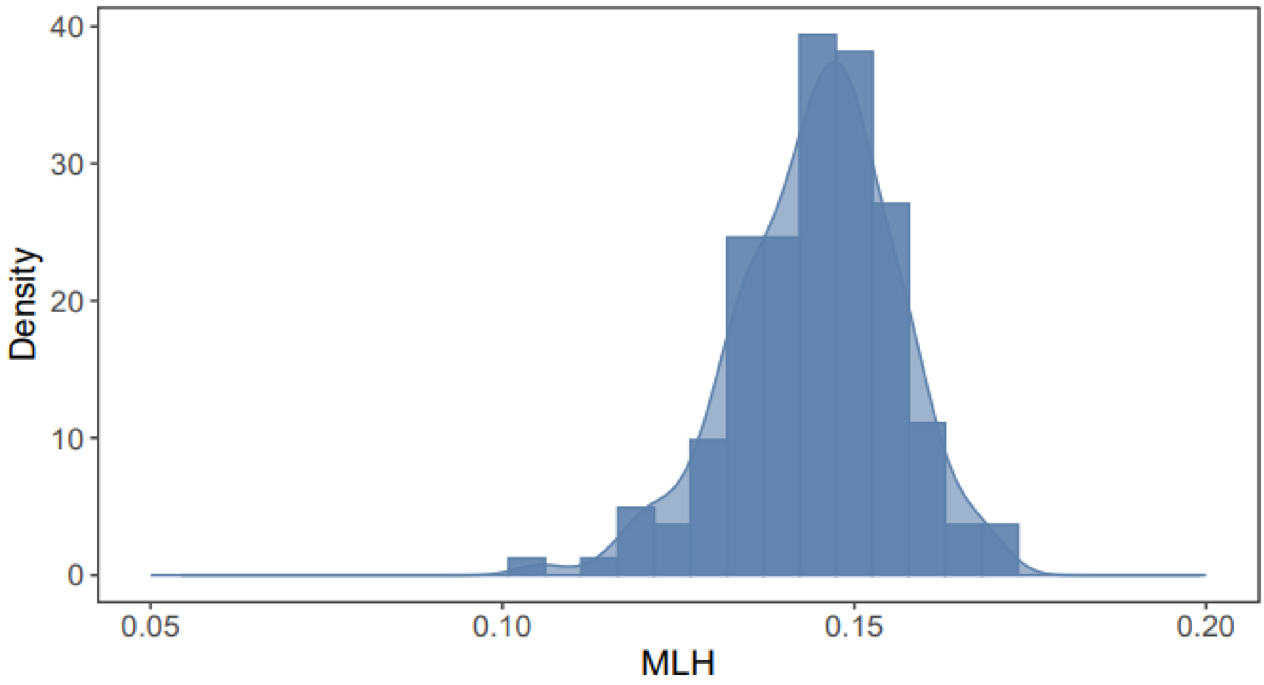

Figure S9: Distribution of multilocus heterozygosity across 157 individuals.

Table *S*1: Estimated migration rates and 95% confidence intervals (in brackets) calculated by BA3-SNPs between all assessed regions and separately between North Se*a* and western Scotland/Ireland. Within-group comparisons are printed in bold.

	Migration into				
From	BAS	WSI	ICE	NS	WNA
BAS	**0.689438 [0.674695, 0.70418]**	0.023238 [0.008209, 0.038266]	0.22145 [0.194121, 0.248779]	0.043625 [0.023971, 0.063279]	0.022225 [0.007829, 0.036621]
WSI	0.015225 [0.008425, 0.022025]	**0.675838 [0.670528, 0.681147]**	0.006475 [0.002109, 0.010841]	0.295988 [0.285628, 0.306347]	0.006525 [0.002107, 0.010943]
ICE	0.014563 [0.004887, 0.024238]	0.014525 [0.004815, 0.024235]	**0.930063 [0.910521, 0.949604]**	0.0265 [0.013221, 0.039779]	0.014338 [0.004809, 0.023866]
NS	0.0112 [0.006817, 0.015583]	0.057688 [0.043369, 0.072006]	0.014938 [0.009896, 0.019979]	**0.911988 [0.896656, 0.927319]**	0.0042 [0.001506, 0.006894]
WNA	0.041525 [0.016033, 0.067017]	0.041263 [0.015917, 0.066608]	0.166888 [0.128064, 0.205711]	0.041913 [0.016299, 0.067526	**0.708413 [0.682946, 0.733879]**

	Migration into	
From	WSI	NS
WSI	**0.99272 [0.986497, 0.998943]**	0.00728 [0.001057, 0.013503]
NS	0.32646 [0.320552, 0.332368]	**0.67354 [0.667632, 0.679448]**

Table S2: Pairwise fixation indices between ten sampled regions. Values in bold are significant (p<0.05).

	**BAS**	**W_SCOT**	**ICE**	**E_SCOT**	**DEN**	**IRE**	**GER**	**ENG**	**NEL**	**WNA**
**BAS**	-									
**W_SCOT**	**0.049**	-								
**ICE**	0.002	**0.062**	-							
**E_SCOT**	**0.028**	**0.005**	**0.043**	-						
**DEN**	**0.029**	**0.013**	**0.038**	0.002	-					
**IRE**	**0.077**	0.025	**0.093**	**0.027**	**0.045**	-				
**GER**	**0.037**	**0.015**	**0.045**	0.004	0.001	**0.048**	-			
**ENG**	**0.031**	**0.005**	**0.044**	0.000	0.000	**0.028**	-0.001	-		
**NEL**	**0.036**	0.006	**0.053**	0.003	-0.002	**0.057**	0.005	-0.003	-	
**WNA**	**0.029**	**0.079**	0.014	**0.056**	**0.059**	**0.123**	**0.071**	**0.060**	**0.073**	-

Table S3: Metadata for each sequenced *Lagenorhynchus albirostris* sample with information on sample date, geographic information, and sex of the animal.

**ID**	**Date**	**Location**	**Latitude**	**Longitude**	**Sex**
WBD100	17.06.2002	Scotland	57.34450531	-1.938509345	F
WBD104	07.06.1999	Barents Sea	76.2	14.31666667	*NA*
WBD107	*NA*	Barents Sea	77.66666667	10.35	*NA*
WBD10	23.07.2005	Scotland	58.59729767	-3.526139021	M
WBD112	22.07.2000	Barents Sea	73.45	36.73333333	M
WBD113	23.07.2000	Barents Sea	71.25	28.8	F
WBD116	30.07.2000	Barents Sea	71.4	33.23333333	F
WBD118	30.07.2000	Barents Sea	71.43333333	30.55	M
WBD119	05.08.2000	Barents Sea	71.81666667	30.3333333	M
WBD11	17.06.1994	Scotland	58.07188416	-5.452246666	F
WBD122	11.08.2000	Barents Sea	74.23333333	21.6	*NA*
WBD123	11.08.2000	Barents Sea	74.25	21.16666667	M
WBD127	10.07.2003	Barents Sea	73.05	10.11666667	M
WBD128	30.03.1994	Iceland	64.57	-23.131	F
WBD129	19.03.1996	Iceland	64.05	-15.4	M
WBD12	09.09.1993	Scotland	57.1521225	-2.77656269	M
WBD130	09.05.1992	Iceland	63.58	-22.48	M
WBD131	26.01.1996	Iceland	63.19	-19.47	F
WBD132	09.10.1995	Iceland	64.16	-22.23	M
WBD133	14.04.1991	Iceland	64.19	-22.18	F
WBD134	08.04.1992	Iceland	63.49	-22.8	M
WBD135	07.04.1992	Iceland	63.5	-21.25	F
WBD136	31.01.1996	Iceland	64	-23.0	F
WBD137	07.04.1992	Iceland	63.5	-21.25	F
WBD138	07.04.1992	Iceland	63.48	-20.59	M
WBD139	03.04.1993	Iceland	64.08	-22.47	F
WBD13	25.08.2018	Scotland	59.1199913	-3.881567	M
WBD140	14.02.1996	Iceland	64.2	-22.54	M

WBD141	1996	Iceland	63.58	-22.5	M
WBD142	28.02.1996	Iceland	63.58	-22.47	F
WBD144	04.04.1992	Iceland	63.51	-21.11	M
WBD145	04,04.1992	Iceland	63.51	-21.11	M
WBD14	11.09.2018	Scotland	58.60188675	-3.357674122	M
WBD151	29.01.1997	Denmark	57.75166667	10.62111	F
WBD152	01.12.1999	Denmark	57.00666667	8.435278	M
WBD153	20.05.1999	Denmark	55.48916667	8.267778	F
WBD154	31.03.1999	Denmark	57.745	10.55806	F
WBD155	25.09.2005	Denmark	57.0776	9.967683	F
WBD156	02.03.2008	Denmark	55.27348833	8.520153	M
WBD157	03.03.2006	Ireland	52.29	-10.04	M
WBD15	05.03.2017	Scotland	59.05299377	-3.337082386	M
WBD161	06.05.1993	Ireland	52.22906	-9.90115	F
WBD162	06.05.1993	Ireland	52.22906	-9.90115	F
WBD165	2003	Germany	54.978751	8.332604	F
WBD166	1993	Germany	54.978751	8.332604	F
WBD167	2010	Germany	54.978751	8.332604	F
WBD168	2006	Germany	54.978751	8.332604	F
WBD169	1998	Germany	54.978751	8.332604	F
WBD16	06.03.2013	Scotland	57.72277778	-3.28	M
WBD170	NA	Germany	54.978751	8.332604	F
WBD171	1993	Germany	54.978751	8.332604	F
WBD172	NA	Germany	54.978751	8.332604	M
WBD173	1992	Germany	54.978751	8.332604	F
WBD174	1990	Germany	54.978751	8.332604	F
WBD175	2007	Germany	54.978751	8.332604	M
WBD176	1993	Germany	54.978751	8.332604	F
WBD177	04.09.1991	England	54.24392869	-0.357803	F
WBD178	30.09.1991	England	53.72803062	0.043492	M
WBD17	20.04.2013	Scotland	57.40388889	-6.492777778	F
WBD182	03.07.1993	England	53.92424514	-0.164753	F
WBD184	25.05.1994	England	54.68924662	-1.193236	F
WBD185	03.07.1994	England	50.24999222	-3.166692	F
WBD186	22.02.1995	England	54.0746023	-0.19726	M
WBD187	16.06.1995	England	52.80755025	1.571738	M
WBD188	20.09.1995	England	55.0028139	-1.409764	F
WBD189	31.12.1995	England	52.20724804	1.625842	F
WBD18	27.07.2004	Scotland	58.60641479	-3.354409218	M
WBD191	06.08.1998	England	55.09255663	-1.479744	F
WBD192	04.12.1999	England	53.5679261	0.0992	F
WBD193	27.02.2004	England	54.58690709	-0.972401	M
WBD194	20.06.2010	England	54.745	-1.269167	M
WBD195	27.01.2011	England	55.2425	-1.547778	F
WBD196	27.07.2011	England	55.30888889	-1.553333	M
WBD198	22.03.2012	England	51.3875	1.3625	M
WBD199	03.05.2012	England	51.365	1.028333	M
WBD19	07.01.2013	Scotland	56.00704956	-2.893220901	F
WBD1	07.10.2019	Scotland	57.38916667	-1.863333333	F
WBD200	10.07.2012	England	52.38055556	1.716667	M
WBD201	14.12.2012	England	54.20833333	-0.281944	M

WBD202	04.01.2018	England	50.65222222	-1.154722	F
WBD203	23.11.2018	England	50.80222222	0.341389	F
WBD204	06.02.2020	England	54.621884	-1.00000	F
WBD205	09.02.2020	England	55.472285	-1.591469	M
WBD206	03.02.2008	Netherlands	51.446	3.644	F
WBD207	26.12.2009	Netherlands	51.973	3.968	F
WBD208	27.12.2009	Netherlands	53.461	5.972	F
WBD209	27.12.2009	Netherlands	53.461	5.972	F
WBD210	15.02.2010	Netherlands	51.633	3.689	F
WBD213	03.01.2012	Netherlands	51.502	3.473	M
WBD214	08.12.2017	Netherlands	52.495	4.582	M
WBD215	08.07.2019	Netherlands	52.071	4.219	F
WBD216	2013	England	*NA*	*NA*	*NA*
WBD218	30.06.2021	Canada	46.53675	-56.36978	F
WBD224	14.06.2021	Canada	46.83675	-56.16978	F
WBD228	27.06.2020	Canada	46.31925	-56.44943	M
WBD23	02.03.2008	Scotland	58.48596191	-5.113494873	F
WBD24	28.05.2005	Scotland	56.36075592	-2.805977106	F
WBD25	16.10.2003	Scotland	57.16739655	-2.77673197	M
WBD26	21.12.2018	Scotland	56.55148697	-2.60182333	M
WBD27	22.12.1992	Scotland	56.21850586	-2.706240654	M
WBD28	30.09.2011	Scotland	59.05209732	-3.337056398	M
WBD2	01.11.2016	Scotland	60.30310059	-1.648934484	F
WBD33	01.09.2016	Scotland	57.586336	-6.3755252	M
WBD34	17.12.2015	Scotland	57.22513962	-5.641182423	M
WBD35	29.12.2015	Scotland	58.19838333	-6.208683491	F
WBD36	13.06.2016	Scotland	58.60824203	-3.351047516	M
WBD37	25.11.2015	Scotland	58.58374786	-4.767692089	F
WBD38	29.07.2016	Scotland	58.59537506	-3.378072262	M
WBD39	13.12.2015	Scotland	56.25305939	-2.631024361	M
WBD3	11.08.2017	Scotland	58.89316559	-2.921369314	M
WBD40	08.07.2015	Scotland	58.84124756	-2.895726204	M
WBD41	22.01.2008	Scotland	57.34706497	-1.93350482	F
WBD42	02.12.2005	Scotland	59.27907944	-2.958089352	M
WBD43	14.02.2004	Scotland	57.68662262	-1.968093753	M
WBD44	16.01.2020	Scotland	57.21888889	-7.425277778	F
WBD47	13.01.2003	Scotland	55.98091507	-2.90384078	F
WBD48	12.12.1997	Scotland	59.04934692	-3.342164993	M
WBD4	27.10.2016	Scotland	58.91473007	-2.783025026	M
WBD51	22.03.2014	Scotland	57.82973099	-4.7207394	F
WBD52	15.10.2014	Scotland	56.70484924	-2.452369213	M
WBD53	26.11.2009	Scotland	57.89355087	-4.571394	M
WBD54	07.11.2009	Scotland	56.36338806	-2.815741062	F
WBD55	11.07.2012	Scotland	57.66908264	-2.348642111	F
WBD56	20.02.1999	Scotland	58.59742355	-3.51581192	M
WBD57	14.08.2017	Scotland	59.12411499	-3.320644379	F
WBD58	11.10.2017	Scotland	58.97109604	-3.353046894	F
WBD59	28.07.2017	Scotland	57.3264122	-1.968409657	F
WBD5	25.06.2008	Scotland	56.5369873	-2.625984192	M
WBD60	03.11.2017	Scotland	57.30653763	-7.400987625	*NA*
WBD61	09.11.2012	Scotland	56.52103424	-6.806591034	M

WBD63	01.10.2012	Scotland	57.51470566	-4.150165558	F
WBD66	02.06.2002	Scotland	57.32730865	-1.966754079	M
WBD67	02.07.2000	Scotland	58.50886536	-3.130962849	F
WBD68	17.07.2001	Scotland	57.32910538	-1.960098147	F
WBD69	12.07.2002	Scotland	55.98553848	-3.291846991	M
WBD6	30.10.2017	Scotland	57.68124771	-2.952453136	M
WBD71	23.08.1996	Scotland	56.03731918	-3.691642046	M
WBD72	13.01.2010	Scotland	59.92186356	-1.293459892	M
WBD73	19.06.2010	Scotland	56.04517746	-2.836317539	F
WBD74	21.12.2009	Scotland	57.87271118	-4.16355515	F
WBD75	19.11.2011	Scotland	56.58779144	-2.51935339	M
WBD76	24.02.2009	Scotland	55.97631073	-2.421403885	F
WBD77	04.12.2001	Scotland	57.68082428	-2.325282812	M
WBD78	06.12.2001	Scotland	58.60691071	-3.54893136	M
WBD79	22.09.2001	Scotland	59.13754654	-3.324640274	F
WBD7	16.06.2014	Scotland	58.59729004	-3.367833853	M
WBD80	11.06.1998	Scotland	60.66934967	-1.68511486	M
WBD81	04.10.2006	Scotland	58.22737503	-6.336555004	F
WBD82	28.07.2015	Scotland	58.57080841	-4.742587566	M
WBD83	17.12.2015	Scotland	56.02324677	-2.588768959	M
WBD84	14.10.2015	Scotland	59.1348381	-3.326295853	M
WBD85	31.07.2005	Scotland	58.53165436	-4.210379601	M
WBD86	01.09.1992	Scotland	57.22438049	-5.636134148	M
WBD89	19.01.1992	Scotland	56.68667984	-5.935827255	F
WBD90	08.03.1993	Scotland	57.14056778	-2.74369431	F
WBD92	19.07.1994	Scotland	57.24838638	-2.34805298	M
WBD93	05.04.1995	Scotland	57.69937134	-2.13426781	F
WBD94	10.09.1995	Scotland	58.49624634	-4.668435097	F
WBD95	16.09.1995	Scotland	57.78640747	-3.855320454	M
WBD96	07.06.1996	Scotland	58.46240234	-5.56893349	F
WBD98	05.07.2000	Scotland	57.42830658	-6.30221462	F
WBD9	25.08.2018	Scotland	55.981884	-3.298130274	F

References

Manichaikul, Ani, Josyf C. Mychaleckyj, Stephen S. Rich, Kathy Daly, Michèle Sale, and Wei-Min Chen. 2010. 'Robust Relationship Inference in Genome-Wide Association Studies'. *Bioinformatics* 26 (22): 2867–73. https://doi.org/10.1093/bioinformatics/btq559.
